# Contribution of both olfactory and systemic pathways for brain targeting of nimodipine-loaded lipo-pluronics micelles: *in vitro* characterization and *in vivo* biodistribution study after intranasal and intravenous delivery

**DOI:** 10.1080/10717544.2016.1236848

**Published:** 2017-02-03

**Authors:** Hassan M. Rashed, Rehab N. Shamma, Emad B. Basalious

**Affiliations:** 1 Department of Labeled Compounds, Hot Labs. Center, Egyptian Atomic Energy Authority, Cairo, Egypt and; 2 Department of Pharmaceutics and Industrial Pharmacy, Faculty of Pharmacy, Cairo University, Cairo, Egypt

**Keywords:** Nimodipine, lipo-pluronic micelles, radiolabeling, subarachnoid hemorrhage

## Abstract

Nimodipine (NM) is the only FDA-approved drug for treating subarachnoid hemorrhage induced vasospasm. NM has poor oral bioavailability (5–13%) due to its low aqueous solubility, and extensive first pass metabolism. The objective of this study is to develop radiolabeled NM-loaded LPM and to test its ability prolong its circulation time, reduce its frequency of administration and eventually target it to the brain tissue. NM was radiolabeled with ^99m^Tc by direct labeling method using sodium dithionite. Different reaction conditions that affect the radiolabeling yield were studied. The *in vivo* pharmacokinetic behavior of the optimum NM-loaded LPM formulation in blood, heart, and brain tissue was compared with NM solution, after intravenous and intranasal administration. Results show that the radioactivity percentage (%ID/g) in the heart of mice following administration of ^99m^Tc-NM loaded LPM were lower compared with that following administration of ^99m^Tc-NM solution, which is greatly beneficial to minimize the cardiovascular side effects. Results also show that the %ID/g in the blood, and brain following intravenous administration of ^99m^Tc-NM-loaded LPM were higher at all sampling intervals compared with that following intravenous administration of ^99m^Tc-NM solution. This would be greatly beneficial for the treatment of neurovascular diseases. The drug-targeting efficiency of NM to the brain after intranasal administration was calculated to be 1872.82%. The significant increase in drug solubility, enhanced drug absorption and the long circulation time of the NM-loaded LPM could be promising to improve nasal and parenteral delivery of NM.

## Introduction

Nimodipine (NM), a 1,4-dihydropyridine calcium channel blocker, has been shown to dilate cerebral arterioles and increase cerebral blood flow, thus has been reported to possess beneficial activity in the treatment of several cerebrovascular disorders, such as cerebrovascular spasm, stroke, and migraine. The therapeutic use of NM includes treatment of subarachnoid hemorrhage, focal or global ischemia, and epilepsy. NM is the only FDA-approved drug for treating subarachnoid hemorrhage-induced vasospasm. National and international guidelines recommend the administration of 60 mg of oral NM every 4 h in patients suffering from subarachnoid hemorrhage (Connolly et al., 2012; Steiner et al., 2013). A well-known and important side effect of NM is a significant reduction in the mean arterial pressure and cerebral perfusion pressure (Diringer et al., 2011; Sandow et al., 2016). Oral administration of NM has several limitations and disadvantages. Being a Class II drug (according of the Biopharmaceutical Classification System), NM suffers from a problem of poor aqueous solubility (3.86 μg/mL). NM also suffers from limited oral bioavailability (5–13%) and clinical efficacy as a result from extensive first pass metabolism in the liver. Thus, intravenous administration is the most common route for NM in case of emergency. However, intravenous administration of NM has several problems. Hypotension, bradycardia, arrhythmias, and eventually death may occur when NM is given parenterally at high doses. Commercial NM injection is prepared by solubilizing NM with 40% solvent mixture: 23.7% (v/v) ethanol and 17% (v/v) PEG 400. According to the intravenous dosage regimen, this formulation requires a time-consuming administration (about 10 h by infusion pump) (Xiong et al., 2008). Moreover, NM ethanol injections may cause local adverse reactions at the administration site, such as pain and inflammation (Soliman et al., 2010). In addition, crystallization caused by the poor water solubility of NM can also occur when the NM injection is diluted by injection solutions, which is dangerous to patients (Song et al., 2012). Consequently, the application of these NM commercial injections has been limited by their high medication cost, low patient compliance, and safety considerations.

As an alternative approach, few attempts were investigated to promote the intravenous delivery of NM via encapsulation in suitable nanosized mixed micelles (Song et al., 2012). The feasibility of long-term, continuous, intra-arterial NM treatment and its effects on macrovasospasm, autoregulation parameters and outcome were evaluated in patients with refractory severe macrovasospasm (Hockel et al., 2016).

In fact, novel formulations which promote safe as well as better clinic application of NM are urgently needed. Thus, there is a pressing need to develop a safe, injectable formulation for NM by means of pharmaceutics. Pluronics are triblock copolymers of poly(ethylene oxide) (PEO) and poly(propylene oxide) (PPO) with the structure PEO–PPO–PEO. Pluronics show low cytotoxicity and weak immunogenicity after topical and systemic administration although they are not biodegradable polymers. This may be due to having molecular weight less than 15 kDa which could be easily filtered and cleared by the kidney (Huang et al., 2009). Pluronic micelles have a core–shell structure with hydrophobic core acting as solubilization depot for the non-polar drugs and hydrophilic corona preventing aggregation, protein adsorption, and recognition by the reticulo-endothelial system, which leads to longer blood circulation time. Also, the small size of polymeric micelles (<100 nm) could increase blood circulation time due to escaping from capturing by the mononuclear phagocytic system in the liver and bypassing the filtration of inter-endothelial cells in the spleen (El-Dahmy et al., 2014).

In our previous study, NM-loaded Lipo-Pluronic micelles (LPM) were successfully prepared, and tested for brain targeting via the oral route compared with the oral solution (Basalious & Shamma, 2015). The objective of this study is to develop radiolabeled NM-loaded LPM and to test its ability to prolong its circulation time, reduce its frequency of administration and eventually target it to the brain tissue. NM-loaded LPM were evaluated for drug payload, solubilization efficiency (SE), micelles size, and zeta potential. The *in vivo* pharmacokinetic behavior of the optimum LPM formulation in blood and brain tissue was compared with NM solution.

## Material

NM was kindly donated by Marcyl for pharmaceutical industries (Cairo, Egypt). Difunctional block copolymers of ethylene oxide/propylene oxide [Pluronic® F127 and Pluronic®P123] were purchased from Sigma chemicals company (St. Louis, MO). l-α-Phosphatidylcholine from soybean was purchased from MP Biomedicals (Santa Ana, CA). Ethanol and Polysorbate 80 were from El-Nasr Chemical Co. (Cairo, Egypt). Technetium-99m (^99m^Tc) was eluted as ^99m^TcO_4_ from ^99^Mo/^99m^Tc generator, Monrol Company, Kocaeli, Turkey. All the materials were used as received without any further modifications.

## Methods

### Preparation of ^99m^Tc-NM complex

NM was radiolabeled with ^99m^Tc (*t_1/2_*_ _=6 h) by direct labeling method using sodium dithionite (Na_2_S_2_O_4_) as the reducing agent to overcome colloidal stannic oxide interference with results of biodistribution in case of using stannous chloride as the reducing agent (Qi et al., 1996; Geskovski et al., 2013). Different reaction conditions that affect radiolabeling yield were studied such as sodium dithionite amount, NM amount, reaction pH, temperature, and time. Experiments of each factor were repeated three times and data were statistically examined using one way ANOVA test. Results for *p* are reported and all the results are given as mean ± SD. The level of significance was set at *p *<* *0.05.

In a 10 ml vial, 0.5 mL of NM solution in absolute ethanol containing 0.3–3 mg of NM was added. Then, 0.5 mL of freshly prepared Na_2_S_2_O_4_ solution in distilled water containing 10–200 mg of Na_2_S_2_O_4_ was added. Then, 100 μL of freshly eluted ^99m^Tc (7.2 MBq) was added to the reaction mixture and pH was adjusted by using different volumes of 0.1 M HCl and/or 0.1 M NaOH solutions. The reaction mixture was shaken by electrical vortex and left at different temperatures for different time intervals before calculating the radiochemical yield. Ascending paper chromatography (PC), thin-layer chromatography (TLC), and high-performance liquid chromatography (HPLC) were used to determine the radiochemical yield of ^99m^Tc-NM.

### Radiochemical yield assay

#### PC and TLC analysis

PC and TLC were used to determine the radiochemical yield and the *in vitro* stability of ^99m^Tc-NM complex. Two different mobile phases were used to determine the percent of ^99m^Tc-NM, free ^99m^TcO_4_^−^ and reduced hydrolyzed ^99m^TcO_2_ colloid (Hall et al., 1998; Motaleb, 2007). Acetone was used as a mobile phase to determine free ^99m^TcO_4_ which moved with the solvent front (*R*_f_ = 1), while ^99m^Tc-NM and reduced hydrolyzed technetium colloid remained at the origin (*R*_f_ = 0).

A mixture of ethanol:water:ammonium hydroxide (2:5:1, v/v/v) was used as a mobile phase to determine reduced hydrolyzed technetium colloid which remained at the origin (*R*_f_ = 0) while free ^99m^TcO_4_^−^ and ^99m^Tc-NM species migrated with the solvent front (R_f_ = 1).

#### HPLC

To determine the radiochemical purity for ^99m^Tc-NM, HPLC analysis was used. Ten microliter of ^99m^Tc-NM complex was injected into the column (Alphabond RP-C18, 300 × 3.9 mm) and UV spectrophotometer detector (SPD-6A) was adjusted to the wavelength 237 nm. The mobile phase components were a mixture of methanol:acetonitrile:water (35: 38: 27, v/v) and the flow rate was adjusted to 1 mL/min (Shang et al., 2013). Fractions of 1 mL were collected separately using a fraction collector up to 20 mL and counted in a well-type NaI(Tl) detector connected to a single channel analyzer.

### Preparation of NM-loaded LPM

NM-loaded LPM were prepared using the method described by Basalious & Shamma (2015) with modification. Briefly, ^99m^Tc-NM (20 mg), Phosphatidylcholine (66 mg) and Pluronic® (F127 and P123) mixture (200 mg) in the ratio 2:1 were accurately weighed and dissolved in ethanol (10 mL) in a round-bottom 1000 mL flask. Polysorbate 80 (20% of the phosphatidylcholine content) was dissolved in the solution. The solvent was slowly evaporated at 60 °C under reduced pressure using a rotary evaporator (Buchi R-110 Rotavapor, Flawil, Switzerland), revolving at 120 rpm for 1 h until a thin dry film was formed on the inner wall of the flask. The dried film was treated with distilled water (∼9 mL) and the flask was allowed to revolve at a fixed hydration temperature of 60 °C for 30 min under normal pressure. The mixture was then sonicated for 1 min and the volume was adjusted into 10 mL at room temperature (25 °C) to obtain nanocarrier dispersions.

### Determination of particle size (PS), polydispersity index (PDI) of NM-loaded LPM

The size and the size distribution of NM-loaded LPM were determined by the dynamic light scattering method using a Malvern Mastersizer (DLS, Zetasizer Nano ZS, Malvern Instruments, Malvern, UK). The samples were diluted with distilled filtered water before measurement until being translucent. Additionally, PDI was measured to assess the particle size distribution. Three samples for each formula were used for size determination and the average values ± SD were calculated.

### Biodistribution studies

The drug biodistribution and pharmacokinetic studies were conducted in accordance with the guidelines set by the Egyptian Atomic Energy Authority for animal experiments. The protocol of the study was reviewed and approved by the institutional review board; Research Ethics Committee Faculty of Pharmacy, Cairo University (REC-FOPCU) in Egypt. Male Swiss albino mice (20–25 g) were used as animal models. The animals were housed under constant environmental (room temperature 25 ± 0.5 °C relative humidity; 65% with a 12 h on/off light schedule) and nutritional conditions (fed with standard mice diet with free access to water) throughout the experimental period. On the study day, the mice were divided into three groups (21 mice per group). The conscious animals were administered intravenous (IV) ^99m^Tc-NM-loaded LPM (group A), intranasal (IN) ^99m^Tc-NM-loaded LPM (group B) and IV ^99m^Tc-NM aqueous solution (group C) at a NM dose equivalent to 0.016 mg/g body weight. Briefly, 160–200 μL ^99m^Tc-NM LPM (containing about 0.32–0.4 mg of NM) were injected through the tail vein of group A mice. In a parallel line, 160–200 μL of ^99m^Tc-NM LPM containing an equivalent amount of NM were instilled into each nostril of group B mice using a micropipette (200 μL) fixed with low density polyethylene tube having 0.1 mm internal diameter at the delivery site. Besides, nearly 200 μL of ^99m^Tc-NM aqueous solution containing an equivalent amount of NM were IV injected to group C mice. During the IN administration, the mice were held from the back in a slanted position. The formulations were administered at the openings of the nostrils. The procedure was performed gently, allowing the animals to inhale all the preparation (Abd-Elal et al., 2016). At different time intervals (0.25, 0.5, 1, 2, 4, 8, and 24 h), three mice were anesthetized by chloroform. All mice organs/tissues were dissected, washed with normal saline, made free from adhering tissue/fluid, and weighed. A sample of mice blood was separated, weighed, and counted for its radioactivity uptake and the total blood radioactivity level was calculated considering blood to constitute 7% of total mice weight (Ding et al., 2012; Motaleb et al., 2012; Rashed et al., 2014). The radioactivity of each organ/tissue as well as the background was counted in a well-type NaI (Tl) crystal coupled to SR-7 scaler ratemeter (Motaleb et al., 2012; Rashed et al., 2014). Percent injected dose per gram (%ID/g ± SD) in a population of three mice for each time point are reported. Data were evaluated using one-way ANOVA test and the level of significance was set at *p *<* *0.05.

The pharmacokinetic parameters were calculated by Phoenix® WinNonlin® 6.4 (Certara, L.P.) by non-compartment analysis (Abdelbary & Tadros, 2013). The mean NM radioactivity uptake (%ID/g) in blood and brain samples were plotted against time (h) and the maximum concentration of NM uptake (*C*_max_) and the time to reach it is (*T*_max_) were easily recorded. The area-under-the-curve from 0 to 24 h (AUC _(0-24)_, %ID/g), the area-under-the-curve from 0 to infinity, the time to reach half plasma concentration (*t*_1/2_, h), and the mean residence time (MRT, h) were also estimated.

The evaluation of the NM brain targeting after IN application was estimated by using drug targeting efficiency percentage (DTE %) (Abd-Elal et al., 2016). DTE percentages (that represent the time average partitioning ratio) of NM formulations were calculated according to the following equation:

$$\mathrm{DTE}\%=\frac{\mathrm{AUC}\;\mathrm{brain}/\mathrm{AUC}\;\mathrm{blood}\_\mathrm{IN}}{\mathrm{AUC}\;\mathrm{brain}/\mathrm{AUC}\;\mathrm{blood}\_\mathrm{IV}}\times 100$$

where AUC brain is the area under the brain NM concentration–time curve from zero to 24 h and AUC blood is the area under the blood NM concentration–time curve from zero to 24 h.

The data in the form of mean values (±SD) of three determinations were statistically analyzed applying one-way ANOVA using SPSS 17 software (SPSS Inc., Chicago, IL). Post-hoc multiple comparisons were performed using Fisher's least significant difference test and the results were considered significantly different when *p* values were less than 0.05.

## Results and discussion

### Preparation of the ^99m^Tc-NM complex

#### Effect of NM amount

**Figure 1.**
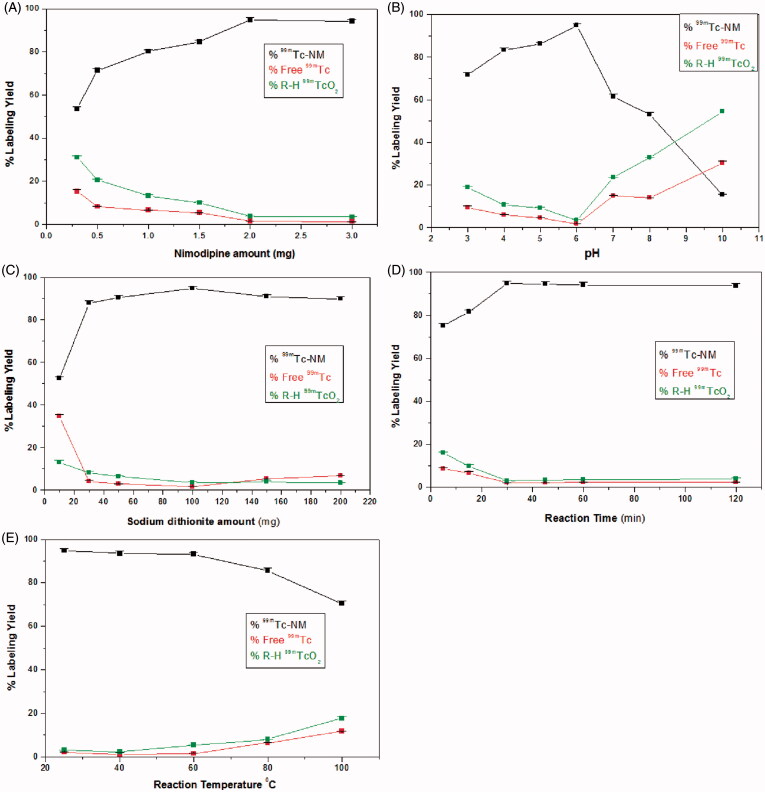
Variation of the radiochemical Yield of ^99m^ Tc-NM as a function of NM amount (A), pH (B), Na_2_S_2_O_4_ amount (C), reaction time (D), and temperature (E).

At 300 μg of NM, the percent labeling yield was low (53.61 ± 0.2%) because NM amount was insufficient to form the complex with all of the reduced technetium (Essa et al., 2015). The maximum radiochemical yield (94.8 ± 0.34%) was obtained by increasing NM amount to 2 mg as shown in Figure 1(A). Increasing NM amount above 2 mg showed no significant change in labeling yield (*p *<* *0.05).

#### Effect of pH of the reaction medium

As shown in Figure 1(B), the maximum radiochemical yield of ^99m^Tc-NM (94.8 ± 0.34%) was obtained at pH 6. The radiochemical yield was very low at highly acidic pH values (71.8 ± 0.58% at pH 3). By increasing the reaction pH above the optimum value, the radiochemical yield was significantly decreased (53.1 ± 0.572% at pH 8). This is attributed to the high OH^-^ concentration at alkaline pH causing partial hydrolysis of the complex and oxidation of ^99m^Tc ^+^ ^5^ to pertechnetate ^99m^TcO_4_^−^ (Amin et al., 2009).

#### Effect of Na_2_S_2_O_4_ amount

A maximum radiochemical yield of 94.8 ± 0.34% was obtained using 100 mg of sodium dithionite as shown in Figure 1(C). At lower amounts of Na_2_S_2_O_4_, high quantities of free pertechnetate were obtained (a labeling yield of 52.33 ± 0.13% at 10 mg) because the used amounts of Na_2_S_2_O_4_ were insufficient for complete reduction of pertechnetate to form ^99m^Tc-complex. Further increase in Na_2_S_2_O_4_ amount above 100 mg leads to labeling yield decrease (90.93 ± 0.31% at 150 mg of Na_2_S_2_O_4_). This is because reduced ^99m^Tc will be slightly re-oxidized when a large excess of dithionite is added resulting in increased level of free ^99m^TcO_4_^−^ (Mayhew, 1978). As noticed, there is no colloidal species other than ^99m^TcO_2_ obtained in the reaction which is a major advantage of using Na_2_S_2_O_4_ as a reducing agent in preparing technetium radiopharmaceuticals avoiding colloidal stannic oxide interference for stannous chloride prepared radiopharmaceuticals (Ferreira et al., 2015; Abd-Elal et al., 2016).

#### Effect of reaction time

As shown in Figure 1(D), short reaction time (5 min) was insufficient to form the ^99m^Tc-NM complex resulting in a low radiochemical yield (75.24 ± 0.61%) (Essa et al., 2015). Increasing the reaction time to 30 min caused an increase in the radiochemical yield to 94.8 ± 0.34%. Further increase of the reaction time over 45 min has no effect on the radiochemical yield.

#### Effect of reaction temperature

Data obtained from this experiment are presented in Figure 1(E). The maximum radiochemical yield of ^99m^Tc-NM was obtained at temperature of 25 ± 2 °C (94.8 ± 0.34%). Increasing the reaction temperature up to about 60 °C did not affect the labeling yield. Further increase to very high temperature (80 and 100 °C) caused a significant decrease of the radiochemical yield due to the increasing the rate of side decomposition reactions at high temperatures (Amin et al., 2009).

#### In vitro stability study of ^99m^Tc-NM

The *in vitro* stability of ^99m^Tc-ligand complexes is essential to determine the suitable time during which the preparation could be used. Decomposition of the ^99m^Tc-ligand complex may occur during storage due to oxidation, hydrolysis, or the radiolysis effect of ionizing γ-radiation (Sakr et al., 2013). ^99m^Tc-NM showed good *in vitro* stability up to 24 h.

### Determination of particle size (PS), polydispersity index (PDI) of NM-loaded LPM

The prepared NM-loaded LPM showed a small particle size of 571.5 ± 11.87 nm with low PDI of 0.43 ± 0.06.

### Biodistribution studies

**Figure 2.**
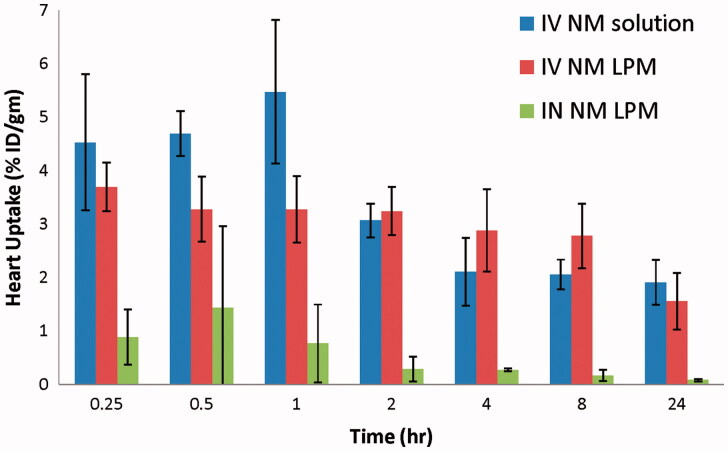
Bar chart showing percentage radioactivity in the heart of mice at different time intervals following administration of radiolabled formulation.

**Figure 3.**
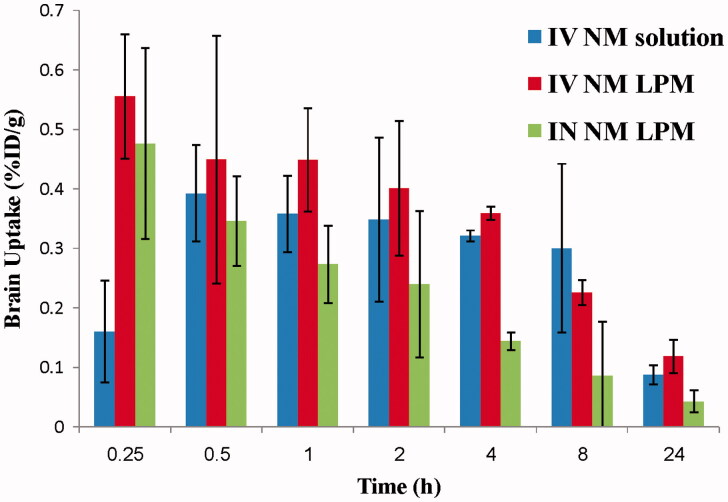
Bar chart showing percentage radioactivity in the brain of mice at different time intervals.

The biodistribution study of IV ^99m^Tc-NM solution, IV ^99m^Tc-NM-loaded LPM, and IN ^99m^Tc-NM-loaded LPM was done on male Swiss albino mice and the radioactivity was estimated at different time intervals up to 24 h as %ID/g for different organs or body fluids. Figures 2 and 3 illustrate the radioactivity (%ID/g) in the heart and brain, respectively, at different time intervals following administration of the radiolabeled formulations.

The pharmacokinetic parameters of all formulations (*C*_max_, *T*_max_, MRT_(0–∞)_, AUC _(0–∞)_, and AUC _(0–24)_) were calculated and displayed in Table 1.

**Table 1.** The mean pharmacokinetic parameters of NM in blood, and brain of mice after administration of single dose of NM solution and NM-loaded LPM via the IV and IN route.

	IN NM-loaded LPM		IV NM-loaded LPM		IV NM solution	
	Blood	Brain	Blood	Brain	Blood	Brain
*C*_max_ (%ID/g)	0.33 ± 0.05	0.51 ± 0.10	24.08 ± 1.73	0.61 ± 0.12	11.30 ± 1.46	0.41 ± 0.09
*T*_max_ (h)	0.58 ± 0.38	0.33 ± 0.14	0.25 ± 0.00	0.33 ± 0.14	0.25 ± 0.00	1.00 ± 0.87
AUC_(0–24)_ (%ID/g h)	2.23 ± 0.20	2.58 ± 0.69	228.91 ± 18.99	5.54 ± 0.23	90.58 ± 8.10	5.60 ± 1.82
*t*_1/2_ (h)	11.17 ± 5.07	10.67 ± 5.24	11.68 ± 1.14	14.00 ± 3.86	13.45 ± 2.37	13.40 ± 6.36
MRT (h)	7.62 ± 1.54	7.76 ± 1.57	8.05 ± 0.49	8.49 ± 0.94	8.57 ± 0.08	8.32 ± 0.80

Results show that the radioactivity percentage (%ID/g) in the heart of mice following IV administration of ^99m^Tc-NM-loaded LPM was lower at the initial sampling intervals, up to 1 h, compared with that following IV administration of ^99m^Tc-NM solution. Moreover, %ID/g in the heart following IN administration of ^99m^Tc-NM-loaded LPM was lower at all the sampling intervals, compared with that following IV administration of ^99m^Tc-NM solution. This would be greatly beneficial for reducing the cardiovascular side effects commonly encountered by parenteral administration of high doses of NM, such as bradycardia, and arrhythmias.

#### Evaluation of IV delivery of NM-loaded LPM (IV LPM versus IV solution)

Results show that the %ID/g in the blood, and brain following IV administration of ^99m^Tc-NM loaded LPM was higher at all sampling intervals compared with that following IV administration of ^99m^Tc-NM solution. This would be greatly beneficial for the treatment of neurovascular diseases. These results could indicate the ability of NM-loaded LPM to achieve IV sustained profile when compared with the IV solution containing the drug in solubilized form. This result suggests that long blood circulation time was achieved by entrapping NM into LPM and thus it can contribute to better clinical efficacy. Long blood circulation time is a critical issue in achieving high targeting accumulation in the brain. These results were in accordance with that previously reported by Wei et al. (2009), who studied the *in vivo* behavior of pluronics mixed micelles after IV injection in rats.

With regard to the extent parameters for NM, ANOVA results indicated significant effects for *C*_max_ and AUC_0–24_ with *p* = 0.012 and *p* = 0.002, respectively. The *C*_max_ in blood following IV administration of ^99m^Tc-NM-loaded LPM, and IV ^99m^Tc-NM solution was significantly higher (about 2-folds) compared to IV 99mTc-NM solution (24.08 ± 1.72 and 11.30 ± 1.46%/g, respectively) (*p *<* *0.05), at the same *T*_max_ (0.25 ± 0.00 h). Moreover, the AUC_(0–∞)_ in the plasma after IV administration of ^99m^Tc-NM-loaded LPM was significantly higher (about 2.5-folds) compared with IV administration of ^99m^Tc-NM solution (228.91 ± 18.98 and 90.58 ± 8.10 h%/g), respectively (*p *<* *0.05). The MRT in the plasma after IV administration of ^99m^Tc-NM-loaded LPM was not significantly changed compared with IV administration of ^99m^Tc-NM solution (8.05 ± 0.48, and 8.57 ± 0.08 h%/g), respectively (*p *>* *0.05).

Study the pharmacokinetic behavior of NM in brain tissue is of great importance as it is the target organ of NM. NM was detected in brain tissues in a significantly higher concentration, as soon as the 15 min sampling time following the administration of the IV administration of ^99m^Tc-NM-loaded LPM, compared with the IV solution, indicating very rapid absorption. The *C*_max_ in brain following IV administration of ^99m^Tc-NM-loaded LPM was significantly higher (about 1.5-folds) (0.61 ± 0.12 and 0.41 ± 0.08%/g, respectively) (*p *<* *0.05), at the significantly shorter *T*_max_ (0.33 ± 0.14 h and 1.00 ± 0.86 h, respectively), compared with that following administration of IV ^99m^Tc-NM solution. On one hand, the high values of *C*_max_ of the LPM compared with that of the solution might be attributed to the long circulation time of the LPM and the presence of Pluronics, polysorbate 80, and phosphatidylcholine in the LPM which acts as a penetration enhancer and inhibitor of P-glycoprotein in the blood–brain barrier (Salama et al., 2012). Similar results were obtained in our previous study (Basalious & Shamma, 2015). On the other hand, the AUC _(0–24)_ in the brain after IV administration of ^99m^Tc-NM-loaded LPM was not significantly changed compared with IV administration of ^99m^Tc-NM solution (5.53 ± 0.23 and 5.60 ± 1.81 h%/g), respectively (*p *>* *0.05). The MRT in the brain after IV administration of ^99m^Tc-NM-loaded LPM was also not significantly changed compared with IV administration of ^99m^Tc-NM solution (8.48 ± 0.94 and 8.32 ± 0.80 h%/g), respectively (*p *>* *0.05).

#### Evaluation of IN delivery of NM-loaded LPM (IN LPM versus IV solution)

**Figure 4.**
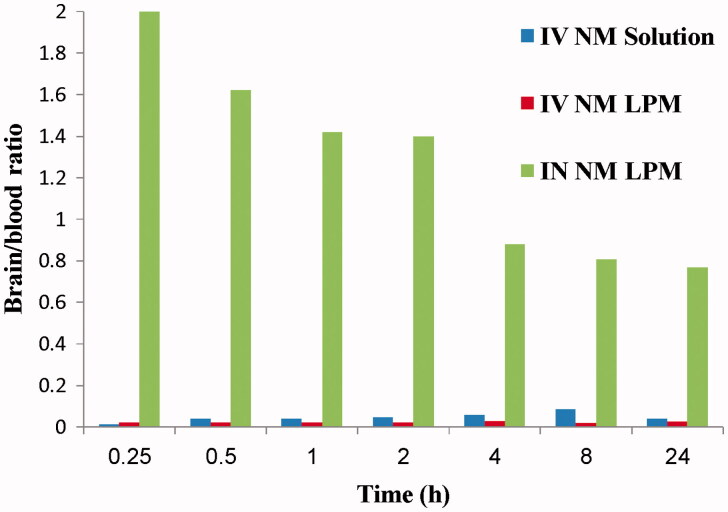
Bar chart showing the brain/blood ratio at different time intervals following administration of radiolabled formulations.

Results show that NM brain peak concentration after IN administration of the ^99m^Tc- NM-loaded LPM was significantly higher than brain NM peak concentration following IV administration of ^99m^Tc-NM solution (*p *<* *0.05). However, the concentration of NM in blood after IN administration was significantly lower than that after IV administration (*p *<* *0.05). Furthermore, the brain/blood ratios and DTE (%) were also estimated. The brain/blood ratios were significantly higher at all sampling intervals in the case of IN administration of ^99m^Tc-NM-loaded LPM compared with that following administration of IV ^99m^Tc-NM solution (Figure 4). The DTE was calculated to be 1872.82%. This indicates that the prepared NM loaded LPM succeeded to penetrate the nasal membrane and deliver 18-folds greater amount of NM at the target site (brain) after IN administration compared with IV administration of the NM solution.

The pharmacokinetic parameters (*C*_max_, *T*_max_, MRT_(0–∞)_, AUC_(0–∞)_, and AUC_(0–24)_) were displayed in Table 1. The *C*_max_ in brain following IN administration of ^99m^Tc-NM-loaded LPM and IV administration of ^99m^Tc-NM solution was 0.513 and 0.406%/g, respectively. This could be explained by the high capability of the lipid nanovesicles to permeate the nasal membrane (Salama et al., 2012; Abdelrahman et al., 2015). This suggests that the nasal route is considered as preferential route for brain targeting than IV route. Lipid nanovesicles have been demonstrated to have good permeability characteristics that enhance nasal penetration of many drugs through disrupting the mucosal membrane, hence increase absorption of drugs (Salama et al., 2012). Intranasal NM-loaded LPM delivery enhanced NM delivery to the brain through the olfactory pathway in which it travels from the nasal cavity to brain tissue (Illum, 2000). The small particle size helped them to squeeze themselves through the small opening in the olfactory neurons to the brain via different endocystic pathways of neuronal cells in nasal membrane (Seju et al., 2011). Similar results were obtained by Kenazawa et al. (Kanazawa et al., 2011), where they reported that NM and coumarin micelle nanocarriers can be delivered to the brain due to their ability to transport trans-cellulary through olfactory membrane.

## Conclusion

NM-loaded LPM was successfully prepared in the nano-size. Systemic and olfactory delivery of ^99m^Tc-NM-loaded LPM showed enhanced brain targeting compared with the IV ^99m^Tc-NM solution. Thus, NM-loaded LPM may be a promising replacement for the NM injection, which contains the organic solvent ethanol. In conclusion, with remarkable improvement in the bioavailability, NM-loaded LPM to be administered via the IV and IN route may, therefore, constitute an advance in the management of cerebro-vascular disorders.

## References

[CIT0001] Abd-Elal RM, Shamma RN, Rashed HM, Bendas ER. (2016). Trans-nasal zolmitriptan novasomes: in-vitro preparation, optimization and in-vivo evaluation of brain targeting efficiency. Drug Deliv 1–13. Epub 2016/04/30. doi: 10.1080/10717544.2016.1183721 27128792

[CIT0002] Abdelbary GA, Tadros MI. (2013). Brain targeting of olanzapine via intranasal delivery of core-shell difunctional block copolymer mixed nanomicellar carriers: *in vitro* characterization, *ex vivo* estimation of nasal toxicity and *in vivo* biodistribution studies. Int J Pharm 452:300–10 23684658 10.1016/j.ijpharm.2013.04.084

[CIT0003] Abdelrahman FE, Elsayed I, Gad MK, et al. (2015). Investigating the cubosomal ability for transnasal brain targeting: *in vitro* optimization, *ex vivo* permeation and *in vivo* biodistribution. Int J Pharm 490:281–91 26026251 10.1016/j.ijpharm.2015.05.064

[CIT0004] Amin AM, El-Azony KM, Ibrahim IT. (2009). Application of 99Mo/99mTc alumina generator in the labeling of metoprolol for diagnostic purposes. J Labeled Comp Radiopharm 52:467–72

[CIT0005] Basalious EB, Shamma RN. (2015). Novel self-assembled nano-tubular mixed micelles of Pluronics P123, Pluronic F127 and phosphatidylcholine for oral delivery of nimodipine: *in vitro* characterization, *ex vivo* transport and *in vivo* pharmacokinetic studies. Int J Pharm 493:347–56 26241752 10.1016/j.ijpharm.2015.07.075

[CIT0006] Connolly ES Jr, Rabinstein AA, Carhuapoma JR, et al. (2012). Guidelines for the management of aneurysmal subarachnoid hemorrhage: a guideline for healthcare professionals from the American Heart Association/American Stroke Association. Stroke: J Cerebral Circul 43:1711–37 10.1161/STR.0b013e3182587839 22556195

[CIT0007] Ding R, He Y, Xu J, et al. (2012). Preparation and bioevaluation of 99mTc nitrido radiopharmaceuticals with pyrazolo[1,5-a]pyrimidine as tumor imaging agents. Med Chem Res 21:523–30

[CIT0008] Diringer MN, Bleck TP, Claude Hemphill J III, et al. (2011). Critical care management of patients following aneurysmal subarachnoid hemorrhage: recommendations from the Neurocritical Care Society's Multidisciplinary Consensus Conference. Neurocrit Care 15:211–40 21773873 10.1007/s12028-011-9605-9

[CIT0009] El-Dahmy RM, Elsayed I, Elshafeey AH, et al. (2014). Optimization of long circulating mixed polymeric micelles containing vinpocetine using simple lattice mixture design, in vitro and in vivo characterization. Int J Pharm 477:39–46 25290813 10.1016/j.ijpharm.2014.10.003

[CIT0010] Essa BM, Sakr TM, Khedr MA, et al. (2015). (99m)Tc-amitrole as a novel selective imaging probe for solid tumor: in silico and preclinical pharmacological study. Eur J Pharm Sci: Off J Eur Feder Pharm Sci 76:102–9 10.1016/j.ejps.2015.05.002 25956074

[CIT0011] Ferreira DS, Boratto FA, Cardoso VN, et al. (2015). Alendronate-coated long-circulating liposomes containing 99mtechnetium-ceftizoxime used to identify osteomyelitis. Int J Nanomed. 10:2441–50 10.2147/IJN.S76168 PMC4381632 25848262

[CIT0012] Geskovski N, Kuzmanovska S, Simonoska Crcarevska M, et al. (2013). Comparative biodistribution studies of technetium-99 m radiolabeled amphiphilic nanoparticles using three different reducing agents during the labeling procedure. J Labelled Comp Radiopharm 56:689–95 24339006 10.1002/jlcr.3097

[CIT0013] Hall AV, Solanki KK, Vinjamuri S, et al. (1998). Evaluation of the efficacy of 99mTc-Infecton, a novel agent for detecting sites of infection. J Clin Pathol 51:215–19 9659263 10.1136/jcp.51.3.215 PMC500642

[CIT0014] Hockel K, Diedler J, Steiner J, et al. (2016). Long-term, continuous intra-arterial nimodipine treatment of severe vasospasm following aneurysmal subarachnoid hemorrhage. World Neurosurgery 88:104–112 26732964 10.1016/j.wneu.2015.11.081

[CIT0015] Huang SJ, Sun SL, Feng TH, et al. (2009). Folate-mediated chondroitin sulfate-Pluronic 127 nanogels as a drug carrier. Eur J Pharm Sci: Off J Eur Feder Pharm Sci 38:64–73 10.1016/j.ejps.2009.06.002 19540339

[CIT0016] Illum L. (2000). Transport of drugs from the nasal cavity to the central nervous system. Eur J Pharm Sci: Off J Eur Feder Pharm Sci 11:1–18 10.1016/s0928-0987(00)00087-7 10913748

[CIT0017] Kanazawa T, Taki H, Tanaka K, et al. (2011). Cell-penetrating peptide-modified block copolymer micelles promote direct brain delivery via intranasal administration. Pharm Res 28:2130–9 21499835 10.1007/s11095-011-0440-7

[CIT0018] Mayhew SG. (1978). The redox potential of dithionite and SO2-from equilibrium reactions with flavodoxins, methyl viologen and hydrogen plus hydrogenase. Eur J Biochem 17:535–47 10.1111/j.1432-1033.1978.tb12269.x 648533

[CIT0019] Motaleb MA, El-Kolaly MT, Rashed HM, Abd El-Bary A. (2012). Radioiodinated paroxetine, a novel potential radiopharmaceutical for lung perfusion scan. J Radioanal Nucl Chem 292:629–35

[CIT0020] Motaleb MA. (2007). Preparation and biodistribution of 99m Tc-lomefloxacin and 99mTc-ofloxacin complexes. J Radioanal Nucl Chem 272:95–9

[CIT0021] Qi P, Muddukrishna SN, Torok-Both R, et al. (1996). Direct 99mTc-labeling of antibodies by sodium dithionite reduction, and role of ascorbate as a stabilizer in cysteine challenge. Nuclear Med Biol 23:827–35 10.1016/0969-8051(96)00082-0 8940727

[CIT0022] Rashed HM, Ibrahim IT, Motaleb MA, Abd El-Bary A. (2014). Preparation of radioiodinated ritodrine as a potential agent for lung imaging. J Radioanal Nucl Chem 300:1227–33

[CIT0023] Sakr TM, Moustapha ME, Motaleb MA. (2013). 99mTc-nebivolol as a novel heart imaging radiopharmaceutical for myocardial infarction assessment. J Radioanal Nucl Chem 295:1511

[CIT0024] Salama HA, Mahmoud AA, Kamel AO, et al. (2012). Brain delivery of olanzapine by intranasal administration of transfersomal vesicles. J Liposome Res 22:336–45 22881283 10.3109/08982104.2012.700460

[CIT0025] Salama HA, Mahmoud AA, Kamel AO, et al. (2012). Phospholipid based colloidal poloxamer-nanocubic vesicles for brain targeting via the nasal route. Colloids Surf B Biointerfaces 100:146–54 22766291 10.1016/j.colsurfb.2012.05.010

[CIT0026] Sandow N, Diesing D, Sarrafzadeh A, et al. (2016). Nimodipine dose reductions in the treatment of patients with aneurysmal subarachnoid hemorrhage. Neurocritical Care 25:29–39 26690937 10.1007/s12028-015-0230-x

[CIT0027] Seju U, Kumar A, Sawant KK. (2011). Development and evaluation of olanzapine-loaded PLGA nanoparticles for nose-to-brain delivery: in vitro and in vivo studies. Acta Biomater 7:4169–76 21839863 10.1016/j.actbio.2011.07.025

[CIT0028] Shang X, Ma S, Li Z. (2013). Development and validation of a RP-HPLC method for determination of nimodipine in sustained release tablets. J Chem 2013:612082

[CIT0029] Soliman GM, Sharma R, Choi AO, et al. (2010). Tailoring the efficacy of nimodipine drug delivery using nanocarriers based on A2B miktoarm star polymers. Biomaterials 31:8382–92 20691471 10.1016/j.biomaterials.2010.07.039

[CIT0030] Song X, Jiang Y, Ren C, et al. (2012). Nimodipine-loaded mixed micelles: formulation, compatibility, pharmacokinetics, and vascular irritability study. Int J *Nanomedicine 7:3689–99 22888228 10.2147/IJN.S33228 PMC3414212

[CIT0031] Steiner T, Juvela S, Unterberg A, et al. (2013). European Stroke Organization guidelines for the management of intracranial aneurysms and subarachnoid hemorrhage. Cerebrovasc Dis 35:93–112 23406828 10.1159/000346087

[CIT0032] Wei Z, Hao J, Yuan S, et al. (2009). Paclitaxel-loaded Pluronic P123/F127 mixed polymeric micelles: formulation, optimization and in vitro characterization. Int J Pharm 376:176–85 19409463 10.1016/j.ijpharm.2009.04.030

[CIT0033] Xiong R, Lu W, Li J, et al. (2008). Preparation and characterization of intravenously injectable nimodipine nanosuspension. Int J Pharm 350:338–43 17920794 10.1016/j.ijpharm.2007.08.036

